# Artificial Intelligence Identifies an Urgent Need for Peripheral Vascular Intervention by Multiplexing Standard Clinical Parameters

**DOI:** 10.3390/biomedicines9101456

**Published:** 2021-10-13

**Authors:** Kristina Sonnenschein, Stevan D. Stojanović, Nicholas Dickel, Jan Fiedler, Johann Bauersachs, Thomas Thum, Meik Kunz, Jörn Tongers

**Affiliations:** 1 Institute of Molecular and Translational Therapeutic Strategies (IMTTS), Hannover Medical School, 30625 Hannover, Germany; stojanovic.stevan@mh-hannover.de (S.D.S.); fiedler.jan@mh-hannover.de (J.F.); thum.thomas@mh-hannover.de (T.T.); 2 Department of Cardiology and Angiology, Hannover Medical School, 30625 Hannover, Germany; bauersachs.johann@mh-hannover.de (J.B.); joern.tongers@uk-halle.de (J.T.); 3 Chair of Medical Informatics, Friedrich-Alexander University (FAU) of Erlangen-Nürnberg, 91054 Erlangen, Germany; nicholas.dickel@fau.de (N.D.); meik.kunz@fau.de (M.K.); 4 Fraunhofer Institute of Toxicology and Experimental Medicine, 30625 Hannover, Germany; 5 Center for Regenerative Translational Medicine, 30625 Hannover, Germany; 6 Department of Internal Medicine III, Martin-Luther-University Halle-Wittenberg, 06097 Halle, Germany

**Keywords:** artificial intelligence, machine learning, classification model, Random Forest, peripheral artery disease, vascular disease

## Abstract

Background: Peripheral artery disease (PAD) is a significant burden, particularly among patients with severe disease requiring invasive treatment. We applied a general Machine Learning (ML) workflow and investigated if a multi-dimensional marker set of standard clinical parameters can identify patients in need of vascular intervention without specialized intra–hospital diagnostics. Methods: This is a retrospective study involving patients with stable PAD (sPAD, Fontaine Class I and II, *n* = 38) and unstable PAD (unPAD, Fontaine Class III and IV, *n* = 18) in need of invasive therapeutic measures. ML algorithms such as Random Forest were utilized to evaluate a matrix consisting of multiple routinely clinically available parameters (age, complete blood count, inflammation, lipid, iron metabolism). Results: ML has enabled a generation of an Artificial Intelligence (AI) PAD score (AI-PAD) that successfully divided sPAD from unPAD patients (high AI-PAD in sPAD, low AI-PAD in unPAD, cutoff at 50 AI-PAD units). Furthermore, the probability score positively coincided with gold-standard intra-hospital mean ankle-brachial index (ABI). Conclusion: AI-based tools may be promising to enable the correct identification of patients with unstable PAD by using existing clinical information, thus supplementing clinical decision making. Additional studies in larger prospective cohorts are necessary to determine the usefulness of this approach in comparison to standard diagnostic measures.

## 1. Introduction

Peripheral artery disease (PAD) of the lower extremities affects more than 200 million people worldwide and is likely an underrecognized disease, with rising prevalence with aging [1]. PAD is associated with significant morbidity and mortality [2]. Up to 50–60 percent of PAD patients die from cardiovascular causes within 10 years [3]. Up to 30 percent of PAD patients are asymptomatic but still have nearly the same cardiovascular mortality as highly symptomatic PAD patients.

Current clinical methods to diagnose PAD are based on the measurement of ankle-brachial index (ABI) or Doppler ultrasound flow measurements [4]. These methods are very useful for PAD diagnosis but require trained staff in an intra–hospital or ambulant setting.

It would be practical to have a non-invasive tool at disposal that could aid hands-off clinical decision-making, based on existing patient data, perhaps decreasing the workload of hospital staff.

Multiple individual biomarkers of PAD have been proposed. However, single biomarkers often either lack sensitivity/specificity or are not available for wide-spread clinical use. Yet, significant amounts of data are generally readily available as a part of routine clinical and laboratory tests.

The aim of the study was to establish a general workflow to identify discriminative multi-dimensional markers for potential clinical diagnostics of vascular intervention using Machine Learning (ML) approaches. We hypothesized that the utilization of these results can be suitable for artificial intelligence (AI)-based datamining. We have analyzed a cohort of patients with stable PAD (sPAD, Fontaine class I and II) and unstable PAD (unPAD, Fontaine class III and IV). The latter group required invasive therapy per current guidelines. We analyzed the records of these patients, containing laboratory tests commonly used in the workup of cardiovascular patients, and generated a multi-dimensional matrix as input for ML, generating a probability output score. In order to compare the predictions of the AI to the results of gold-standard clinical assessment, we measured the ABI.

## 2. Materials and Methods

### 2.1. Patient Data Collection

This study is a single-center, retrospective analysis of data and blood measurements collected from patients with PAD. PAD patients undergoing angiologic interventions at the Department of Cardiology and Angiology (Hannover Medical School) between 2017–2019 were included. Informed consent was obtained from all subjects involved in the study for the retrospective use of the data. The study was conducted according to the guidelines of the Declaration of Helsinki and approved by the local Ethics Committee of Hannover Medical School (Nr.9548_BO_K_2021, 08.01.2021).

The following clinical data containing laboratory tests were collected: blood count (erythrocytes, platelets, leukocytes, hemoglobin, hematocrit), lipid panel (LDL cholesterol, HDL cholesterol, triglycerides), inflammation (CRP), liver and kidney function tests (GOT, GPT, urea, serum creatinine, estimated glomerular filtration rate eGFR), electrolytes (sodium, potassium) and iron metabolism (serum ferritin, transferrin, transferrin saturation). Additional clinical data was also taken into consideration (age, sex, NYHA Class, systolic blood pressure, smoking status, history of ischemic cardiac disease, medication).

The inclusion criteria for PAD patients were Fontaine stadium I–IV as determined by reduced ABI < 0.9 and arterial Doppler ultrasound or CT angiography. Following patient risk factors were documented: age, sex, arterial hypertension, diabetes mellitus, smoking, obesity, hyperlipidemia, history of heart failure and history of ischemic cardiac disease. Data of clinical presentation, medication, blood values and therapeutic details were recorded and retrospectively analyzed for the current study.

### 2.2. Statistical Analysis and Modeling

Statistical analysis and modeling were performed by R version 4.1.0 (R Foundation for Statistical Computing, Vienna, Austria). Patient characteristics and clinical parameters are shown as frequencies for categorical variables and as means for numerical variables. Groups (sPAD, unPAD) were compared using the Mann–Whitney-U test (False discovery rate (FDR)-adjusted *p*-value < 0.05; method of Benjamini and Hochberg).

Diagnostic markers were calculated using ML methods Random Forest (RF), logistic regression modeling and stepwise regression using R package caret version 6.0.88 (https://doi.org/10.18637/jss.v028.i05; assessed on 17 September 2021). For model generation, we divided the patients into sPAD and unPAD groups, and model performance was evaluated based on predictive power (accuracy). The models were further used to calculate an AI-Score (AI-PAD). The score represents a scale for assessing disease severity between 0 and 100, with higher Fontaine classes being assumed to represent more severe disease. Typically, scores tend to be in the marginal ranges of the scale, i.e., approximately 0–30 or 70–100. The two groups result from the definition of sPAD and unPAD. For an even separation of the groups, the midpoint value of 50 is used as the cutoff. Due to the low sample number limiting data splitting, we performed additional validation steps using spearman correlation (R package Hmisc version 4.5.0; http://biostat.mc.vanderbilt.edu/s/Hmisc; assessed on 17 September 2021) and partial plot analysis (R package randomForest version 4.6.14; https://doi.org/10.1023/A:1010933404324; assessed on 17 September 2021) of the features. Data were visualized using the R packages corrplot version 0.90 (https://github.com/taiyun/corrplot; assessed on 17 September 2021) and ggplot2 version 3.3.5 (https://www.springer.com/gp/book/9783319242750; assessed on 17 September 2021).

Subsequent statistical analysis was conducted in GraphPad Prism Software (Version 9.2.0, GraphPad Software, Inc., San Diego, CA, USA) via the *t*-test and Pearson correlation analysis (*p* < 0.05 considered as statistically significant). The data displayed a normal distribution for the AI-PAD and ABI (Shapiro–Wilk test, *p* > 0.05 for both values in both sPAD and unPAD group).

## 3. Results

### 3.1. Patient Characteristics and Correlation Analysis

We conducted a retrospective study with clinical data (Table 1) obtained from patients belonging to sPAD (Fontaine class I or II) or unPAD (Fontaine class III or IV). unPAD patients required surgical or percutaneous intervention per current guidelines [4]. Patient groups were sex and age matched. Analysis shows no statistically significant differences between the groups (Mann–Whitney-U test, FDR < 0.05, Table 1).

**Figure 1:**
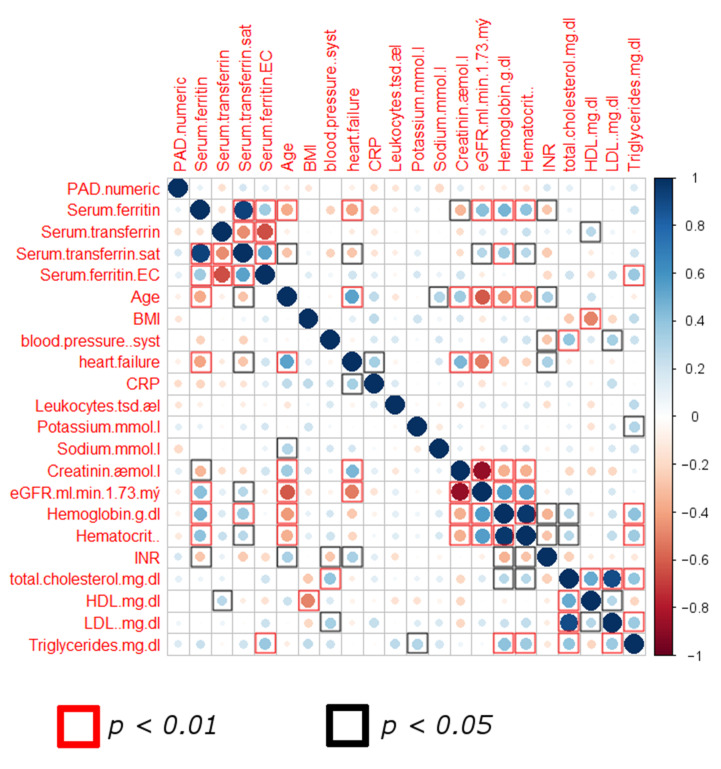
Correlation matrix screening of pooled data from both patient groups. Scale displays Spearman correlation (r) values. (Abbreviations: sat: saturation; BMI: body mass index; CRP: C-reactive protein; INR: International Normalized Ratio; *p*-value < 0.05 as significance level).

We therefore applied additional approaches to investigate a combinatorial effect. An initial screening of the pooled data of all patients showed interrelations between various clinical parameters in a two-dimensional correlation analysis (Spearman test, Figure 1). Individual parameters did not show correlation to PAD stage, thus lacking specificity to identify patients with advanced PAD. However, the majority of parameters showed an interlinkage, displaying statistically significant correlations between values. We thus hypothesized that a more complex computational model could exploit these interconnections via the generation of a decision-tree algorithm that could guide a correct sorting of stable and unstable PAD patients.

### 3.2. ML-Based Scoring for the Identification of Differences between sPAD and unPAD

**Figure 2:**
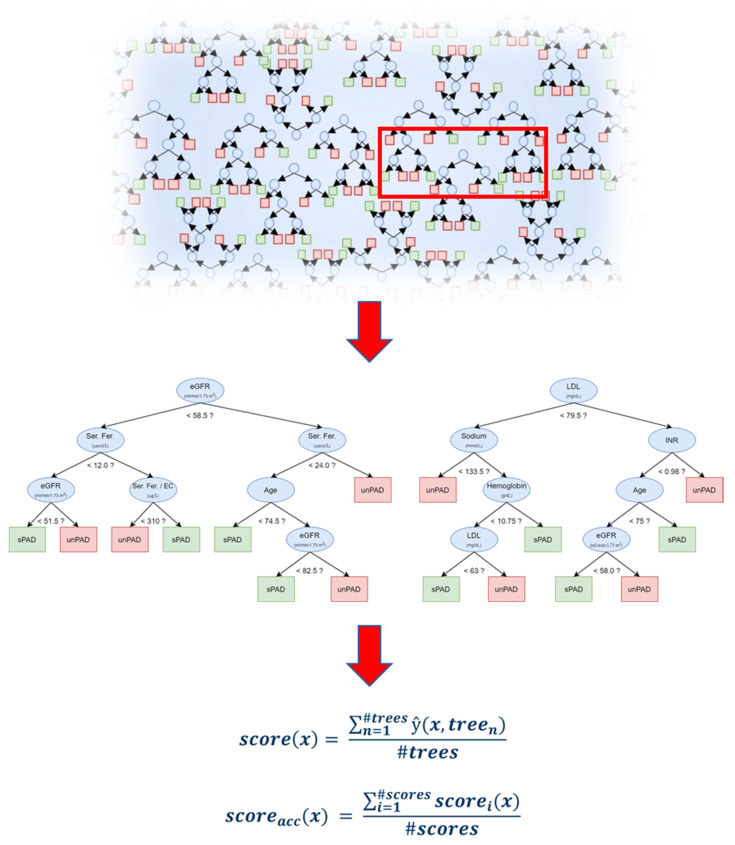
Schematic representation of the Random Forest ML algorithm and formula used to generate the AI-based score (AI-PAD). ‘?’ indicates an exemplary cut-off value (<) generated by the high throughput algorithm.

**Figure 3:**
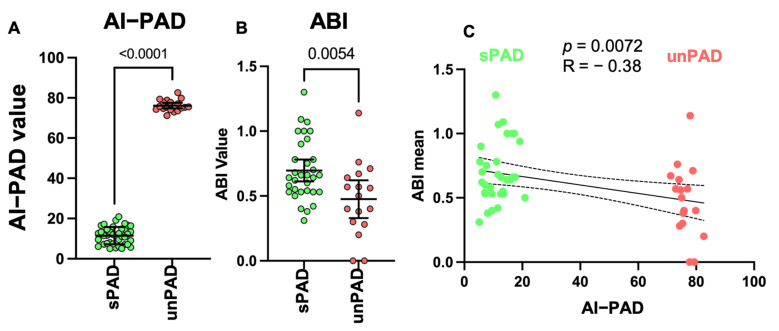
(**A**) AI-PAD in sPAD and unPAD (*t*-test, *p*-value < 0.05 as significance level). (**B**) ABI in sPAD and unPAD (*t*-test, *p*-value < 0.05 as significance level). (**C**) Pearson correlation analysis showing negative ABI-AI-PAD correlation between sPAD and unPAD (*p*-value < 0.05 as significance level).

We further analyzed the data using ML methods RF, logistic regression modeling and stepwise regression, in which RF shows best performance. The developed high-throughput RF model sorting patients into sPAD and unPAD groups is schematically shown in Figure 2. The algorithm generates a quantifiable AI scoring system (AI-PAD, formula shown in Figure 2). AI-PAD showed a significantly higher value amongst unPAD patients, clearly delineating these patients from sPAD (Figure 3A). We proceeded to compare AI-PAD performance to the current gold-standard ABI. ABI values were significantly lower in unPAD patients as expected but displayed a higher level of variability than the AI-PAD (Figure 3B). Nevertheless, a combination of ABI and AI-PAD yielded clear clustering of the sPAD and unPAD group (Figure 3C). Therefore, the AI-PAD showed comparable usefulness to gold-standard ABI measurements in identifying unPAD patients.

## 4. Discussion

Analysis of large datasets with AI may develop into a helpful tool to guide the clinical decision process. The approaches can be adapted into a user-friendly format, requiring input of pre-defined data categories. Such approaches have already garnered interest in radiology, where ML imaging analysis of MRI images was reported to be non-inferior in the recognition of lung cancer, in comparison to experienced physicians [5]. ML improved the prediction capabilities of blood-based biomarker studies, using novel non-coding microRNA molecules [6,7,8]. Further studies show its applicability, for instance in plasma metabolome profiling for the diagnosis of adrenocortical tumours [9].

In order to emulate a realistic clinical scenario, we analysed multiple commonly available clinical markers. Aging, smoking, hypertension, inflammation and the lipid profile are standard components of clinical scoring systems [10,11,12]. We included iron metabolism measurements, due to the rising importance of iron for PAD [13,14].

We could show that the addition of further clinical and laboratory factors to the ABI measurement leads to better identification of patients at risk for advanced PAD. Even without ABI, the AI had significant correlation with gold-standard in-hospital ABI measurement by trained staff and is well-suited to identify undiagnosed PAD patients in a high-risk clinical setting.

The main limitations of this study are the relatively small patient group size and retrospective design, thus generally limiting downstream analysis. Despite the fact that ML-based approaches imply the need of large data cohorts, we decided to utilize them as a starting point for clinical marker evaluation by building AI scoring models in the context of vascular intervention diagnostics. We combat the low sample number limiting data splitting by applying several ML algorithm (RF, logistic regression, stepwise regression) as the basis for AI-PAD calculation, combined with correlation and partial plot analysis as validation steps of the feature selection process [15]. 

The RF model performed best and was therefore used in our pilot study. Which model is ultimately the most suitable always depends on the data and its inherent structure. RF can map a complex structure for prediction by linking several decision trees. Unlike other algorithms (e.g., Elastic Net), RF are not limited by mathematical assumptions such as linearity. Since the individual decisions are always binary, RF can handle both continuous and categorical features. However, alternative algorithms should always be considered for the model generation. This allows to evaluate the similarities and differences between the models, as simpler models can often produce good results and can be used for model generation. 

The identified ML-based scoring system shows the potential to be useful for clinical classification, however prospective studies with more participants would be needed to form a conclusive evaluation of the AI performance, in comparison to classical clinical testing. AI algorithms must be further improved for potential every-day clinical implementation. 

We have conducted a pilot study that serves as a conceptual framework for automatic estimation of disease severity by ML algorithms, which initially eliminates the need for model generalization but becomes important with further application. Therefore, we recommend using proven methods such as data splitting and cross-validation for improved generalization of the models when applying the concept. However, we do not believe that even optimal AI algorithms will soon replace trusted clinical methods such as ABI measurement, but may help with patient screening outside the hospital, decreasing medical staff workload, thus allowing more patient–physician interaction time and better quality of care.

Taken together, we could demonstrate the promise of AI algorithms in identifying patients needing intensive PAD treatment based on basic patient history and standard clinical labs, which should be further evaluated in larger patient cohorts.

## Figures and Tables

**Table 1:** Patient cohort characteristics: sPAD (Fontaine I, Fontaine II) and unPAD (Fontaine III, Fontaine IV) groups. sPAD: stable PAD, unPAD: unstable PAD; *p*-Value < 0.05 as significance level.

	sPAD (*n* = 38)	unPAD (*n* = 18)	*p*-Value	adj. *p*-Value (FDR)
**Sex-no. (%)**				
Male	28 (74)	14 (78)		
Female	10 (26)	4 (22)		
**Age (years)**	70.3	71.1	0.6542	0.7414
**Fontaine Class**				
I	4			
IIa	1			
IIb	33			
III		12		
IV		6		
**Clinical parameters**				
Serum Ferritin (µmol/L)	14.4474	14.6111	0.8535	0.8535
Serum Transferrin (µmol/L)	55.3421	53.0556	0.4392	0.7058
Serum Transferrin Saturated (%)	26.9211	28.4444	0.7188	0.7566
Serum Ferritin/EC (μg/L)	150.4211	226	0.6673	0.7414
BMI (kg/m^2^)	26.6342	26.0111	0.3709	0.6982
Blood Pressure (mmHg)	136.3947	132.3889	0.6356	0.7414
CRP (mg/L)	7.0842	4.0833	0.3569	0.6982
Leukocytes (tsd/µL)	8.2921	7.9056	0.5985	0.7414
Potassium (mmol/L)	4.5105	4.6	0.4588	0.7058
Sodium (mmol/L)	139.4211	138.1111	0.384	0.6982
Creatinin (µmol/L)	128.4474	124.6667	0.2468	0.6982
eGFR (ml/min/1.73 m^2^)	68.3684	59.6111	0.1629	0.6982
Hemoglobin (g/dL)	13.0763	12.6389	0.3344	0.6982
Hematocrit (%)	38.8789	37.3	0.2398	0.6982
INR	1.0366	1.0261	0.06259	0.6982
Cholesterol (mg/dL)	152.3684	159.3889	0.3658	0.6982
HDL (mg/dL)	53.5263	49.9444	0.648	0.7414
LDL (mg/dL)	87.3421	92.7222	0.3045	0.6982
Triglycerides (mg/dL)	127.7105	162.8889	0.1527	0.6982

## Data Availability

All data are available in manuscript and as supplement online.
